# An Analytical Method for Quantifying the Yields of DNA Double-Strand Breaks Coupled with Strand Breaks by γ-H2AX Focus Formation Assay Based on Track-Structure Simulation

**DOI:** 10.3390/ijms24021386

**Published:** 2023-01-10

**Authors:** Yoshie Yachi, Yusuke Matsuya, Yuji Yoshii, Hisanori Fukunaga, Hiroyuki Date, Takeshi Kai

**Affiliations:** 1 Graduate School of Health Sciences, Hokkaido University, Kita-12 Nishi-5, Kita-ku, Sapporo 060-0812, Japan; 2 Faculty of Health Sciences, Hokkaido University, Kita-12 Nishi-5, Kita-ku, Sapporo 060-0812, Japan; 3 Japan Atomic Energy Agency (JAEA), Nuclear Science and Engineering Centre, Research Group for Radiation Transport Analysis, 2-4 Shirakata, Tokai, Naka-gun 319-1195, Japan; 4 Central Institute of Isotope Science, Hokkaido University, Kita-15 Nishi-7, Kita-ku, Sapporo 060-0815, Japan

**Keywords:** Monte Carlo track-structure simulation, complex DSB, γ-H2AX focus formation assay, photon irradiation

## Abstract

Complex DNA double-strand break (DSB), which is defined as a DSB coupled with additional strand breaks within 10 bp in this study, induced after ionizing radiation or X-rays, is recognized as fatal damage which can induce cell death with a certain probability. In general, a DSB site inside the nucleus of live cells can be experimentally detected using the γ-H2AX focus formation assay. DSB complexity is believed to be detected by analyzing the focus size using such an assay. However, the relationship between focus size and DSB complexity remains uncertain. In this study, using Monte Carlo (MC) track-structure simulation codes, i.e., an in-house WLTrack code and a Particle and Heavy Ion Transport code System (PHITS), we developed an analytical method for qualifying the DSB complexity induced by photon irradiation from the microscopic image of γ-H2AX foci. First, assuming that events (i.e., ionization and excitation) potentially induce DNA strand breaks, we scored the number of events in a water cube (5.03 × 5.03 × 5.03 nm^3^) along electron tracks. Second, we obtained the relationship between the number of events and the foci size experimentally measured by the γ-H2AX focus formation assay. Third, using this relationship, we evaluated the degree of DSB complexity induced after photon irradiation for various X-ray spectra using the foci size, and the experimental DSB complexity was compared to the results estimated by the well-verified DNA damage estimation model in the PHITS code. The number of events in a water cube was found to be proportional to foci size, suggesting that the number of events intrinsically related to DSB complexity at the DNA scale. The developed method was applicable to focus data measured for various X-ray spectral situations (i.e., diagnostic kV X-rays and therapeutic MV X-rays). This method would contribute to a precise understanding of the early biological impacts of photon irradiation by means of the γ-H2AX focus formation assay.

## 1. Introduction

**Figure 1:**
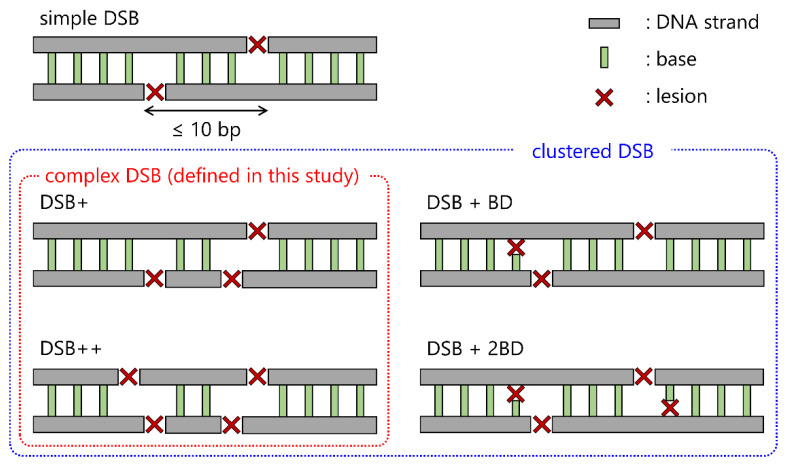
Definition of complex DSBs in this study. This illustration shows the schematic diagram of several types of DSBs. In general, the clustered DSB is a DSB coupled with more than a lesion including strand break and base damage. In this study, we focused on the complex DSB defined to the multiple lesions (only composed of strand breaks) such as DSB+ and DSB++, which is a DSB coupled with one or two strand breaks, respectively.

When the human body is exposed to photon beams, such as X-rays and γ-rays, high-energy photoelectrons are generated after interactions with human tissues (mainly composed of liquid water) [1]. Such photoelectrons induce inelastic interactions, such as ionization and excitation, and deposit their energies into the cell nucleus, inducing DNA damage [2]. There are various types of radiation-induced DNA lesions, including single-strand breaks (SSBs), double-strand breaks (DSBs), and base damage (BD) [3]. Among these, DSBs are recognized as complex lesions which can induce cell death with a certain probability [4,5]. Therefore, when investigating early biological effects, DSB yields and the relative biological effectiveness (RBE) of DSBs are usually evaluated. DSBs can be repaired by virtue of their DNA repair function, i.e., non-homologous end joining (NHEJ) and homologous recombination (HR) [6]. Meanwhile, DSBs coupled with additional strand breaks (SBs) and BD (illustrated in Figure 1) are believed to be refractory damage [7]. Therefore, to evaluate the biological impacts of exposure to ionizing radiation or X-rays, it is necessary to quantify DSB complexity, which can lead to cell killing, using in vitro experiments.

When quantifying DSB yields in vitro, immunofluorescence staining for phosphorylated histone H2AX (γ-H2AX) [8] enables the detection of a DSB site from the focus observable by a fluorescence microscope. This approach is widely used in facilities worldwide in the field of radiation biology [9,10,11]. Using this approach, we investigated the dependency of the RBE for DSBs on photon energy in our previous studies, in which we found that the yields are intrinsically related to the electron track structure [12,13]. Meanwhile, using transmission electron microscopy (TEM) or atomic force microscopy (AFM), a technique for directly detecting clustered DSBs has recently been developed [14,15,16]. Thus, the localization of DNA lesions can be experimentally quantified. However, these microscopies are expensive and available only in limited facilities. As a simple approach, DNA lesion complexity, that is, the site of the DSB coupled with SBs, is sometimes analyzed using the focus size (i.e., area and width) of γ-H2AX [11,17]. However, as the spatial resolution of fluorescence microscopes ranges from several hundred nm to several μm, which does not correspond to the DNA scale (i.e., 10–20 bps (a few nm) [15,18]), the relationship between the foci size and DSB complexity remains uncertain.

To clarify this relationship, we focused on a Monte Carlo (MC) track-structure simulation, which is traditionally used as an effective tool for investigating the mechanisms of DNA damage induction [19]. To date, MC codes such as KURBUC [20], PENELOPE [21,22], Geant4-DNA [23], TOPAS-nBio [24], WLTrack [25], and the Particle and Heavy Ion Transport code System (PHITS) [26] have been developed to simulate the detailed track structure of electrons with kinetic energy down to several eV and atomic interaction at the nm scale [18]. Among these codes, we chose the PHITS electron track structure mode (*etsmode*) [27,28] and an in-house code of WLTrack [25] as we could successfully estimate the DSB yields and the complexity using these codes in our previous studies [12,13,18,29,30]. In particular, the PHITS code is available for any PHITS user free of charge. Considering these, the relationship between the foci size and DSB complexity can be clarified using these simulation tools, leading to the development of an analytical method for complex DSBs applicable to any facility.

In this study, using electron track structure codes (PHITS and WLTrack), we developed an analytical method for quantifying the yield of a complex DSB coupled with additional strand breaks (see Figure 1) from the fluorescence microscopic images of γ-H2AX foci. From the comparison between the estimation of the DSB complexity (i.e., the number of additional SBs at the DSB site) by the MC codes and the foci size, we present an analytical method for complex DSBs that can be applied to X-ray spectral situations (i.e., diagnostic kV X-rays and therapeutic MV X-rays). This developed method would contribute to a precise understanding of the early biological impacts of irradiation from the γ-H2AX focus formation assay.

## 2. Results and Discussion

### 2.1. Spatial Distribution of γ-H2AX Foci and Foci Area

**Figure 2:**
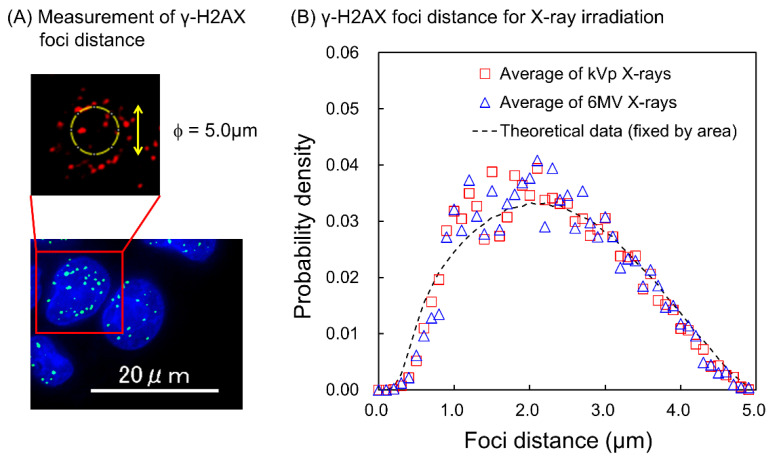
The distance between two γ-H2AX foci induced after X-ray irradiation. (**A**) shows the measurement of γ-H2AX foci distance. The sampling circle with 5 μm diameter is set on the nucleus and γ-H2AX foci distance within the circle were measured (see Section 3.3). (**B**) shows the experimental distributions for 35–150 kVp X-rays and those for 6 MV-linac X-rays (with 1 Gy) are shown as red squares and blue triangles, respectively. Both distributions are in good agreement with the theoretical distribution, which was calculated from two points generated randomly in the circle (black dotted line).

We detected DSB sites in the cell nucleus 30 min after X-ray irradiation using the γ-H2AX focus formation assay. The X-ray energy spectrum used in this study was categorized as diagnostic X-rays (35, 40, 50, 60, 80, 100, 120, and 150 kVp) and therapeutic X-rays (6 MV-linac at 1, 5, and 10 cm depths in water). First, to evaluate the spatial patterns of foci generation in the cell nucleus, we measured the distance between two foci sites in the cases of the diagnostic kVp and therapeutic MV X-rays. Note that we scored the foci distance within a circle with a 5 μm diameter in the cell nucleus (see Figure 2A and Section 3.3). As shown in Figure 2B, the distribution of foci distance for kVp X-rays shows a tendency similar to that for 6 MV X-rays. If the spatial resolution of microscopy is sufficiently high to distinguish multiple DSBs at the nm scale, the combination frequency of short-distance foci is expected to increase, which means that DSBs coupled with SB or DSB within 3.4 nm can be detected. However, as shown in Figure 2B, such a peak was not observed.

We then calculated the theoretical distribution of the distance between the two points generated randomly in the circle (Section 3.4), which is depicted as a dotted line in Figure 2B. The comparison shows that the experimental distributions agreed well with the theoretical distribution. Note that we did not count the simulated distances in the case of a focus piled up with an adjacent focus to consider the detection loss due to spatial resolution in the experiment (see Section 3.4). These results suggest that the spatial patterns of γ-H2AX foci induction after X-ray irradiation are random and independent of X-ray energy. The present result is reasonable as this tendency agrees with the previous report by Löbrich, which showed a random spatial distribution of DSBs after photon irradiation [31].

The development of a super-resolution localization microscope [32] enabled the analysis of DSB sites with a higher spatial resolution (i.e., a few hundred nm) than the microscopy used in this study (i.e., a few μm). However, even when using a super-resolution localization microscope, the minimal distance between two foci was approximately 500 nm, which is not sufficient to detect clustered DSBs coupled with additional damage at the scale of 10–20 bp (corresponding to 3.4–6.8 nm) [15,18]. From these results, we confirmed that DSB complexity cannot be evaluated from the distance and density of foci because of the limitation of the microscopic resolution. Thus, in the next section, we focus on the γ-H2AX foci area (size) as an indicator of DSB complexity.

**Figure 3:**
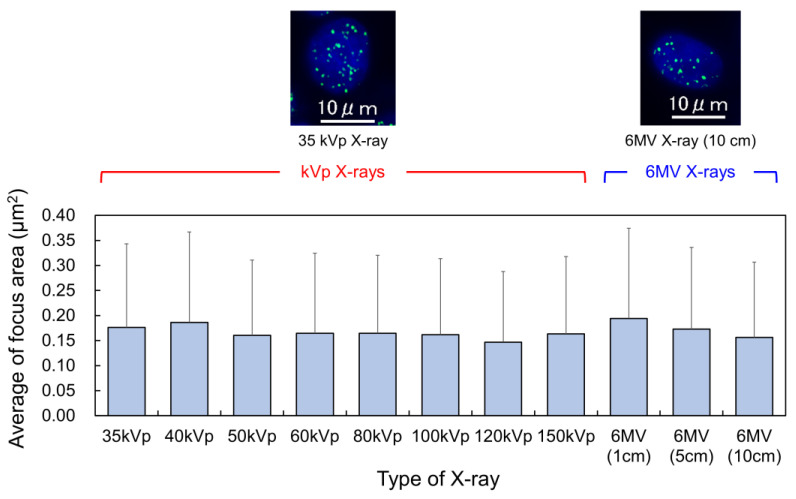
The mean area of γ-H2AX foci induced after various X-ray spectra. The error bar represents the standard deviation (s.d.). In the upper images, green dot and blue area represent DSB site and cell nucleus, respectively. There is no significant difference by the Scheffe’s multiple comparison procedure. From the comparison, it was found that the average of the focus area is independent of the X-ray spectra considered in this study.

Based on the results shown in Figure 2B, we measured the area of the γ-H2AX focus to evaluate the DSB complexity and the dependency of the foci area on photon energy. The averaged areas of foci for various X-ray spectra were compared, as shown in Figure 3. The probability density distribution of the foci area for each X-ray spectra is shown in Figure S1 in the Supplementary Material. The energy spectra and the mean X-ray energy for each X-ray spectra are also summarized in Figure S2 and Table S1 in the Supplementary Material, respectively. There was no significant difference in any of the X-ray energies. This suggests that the yield of complex DSBs is independent of the X-ray spectra. In addition, the number of nuclear γ-H2AX foci depends on X-ray spectra, as shown in our previous study, in which irradiation with kVp X-rays induced more nuclear γ-H2AX foci than irradiation with MV X-rays [12]. This can be explained by the fact that most DSBs are generated at the track end of the photoelectrons [33], and the positions of the track end are relatively located randomly. These results indicate that the secondary electrons ionized by the photoelectrons at the track end play a key role in evaluating DSB induction.

Electrons and photons are categorized as low linear energy transfer (LET) radiation. In the case of high-LET radiation (such as heavy ions), Nakajima et al. and Antonelli et al. showed that the γ-H2AX foci area caused by inducing closely localized individual DSBs instead of clustered DNA damage was larger than that for low-LET radiation [11,17], whereas unchanged averages of the γ-H2AX foci area were observed when using similar LET radiations. For example, Antonelli et al. show the foci area formed after irradiation with α-particle is 1.66 times higher than that irradiated with γ-ray [17]. These results suggest that measuring foci area is an effective approach to evaluate the degree of DNA damage localization depending on LET.

### 2.2. Relationship between γ-H2AX Foci Area and Additional Strand Break Induction

As shown in Figure 2 and Figure 3, we experimentally evaluated the features of the γ-H2AX foci induced 30 min after photon irradiation. Even in this study, the foci area was found to be a candidate for quantifying DSB complexity from foci imaging. Meanwhile, our previous study [13,18] showed that the spatial patterns of events (i.e., ionizations and excitations) play a key role in determining the DSB site and yields of complex DSBs. Here, we estimated the number of events per nano-sized cube (corresponding to the sub-micro-size voxel of foci area) (the so-called cluster size) based on the track-structure simulations (see Section 3.6), and obtained the relationship between the cluster size and the foci area. Because the microdosimetric distributions of photon beams calculated by WLTrack agreed well with those measured by a tissue-equivalent proportional counter (TEPC) [5,12], we adopted WLTrack to analyze the number of events per cube (at the DSB site). Note that the cube size was set to 5.03 × 5.03 × 5.03 nm^3^, which is equivalent to that of the sampling site in a previous study [34].

**Figure 4:**
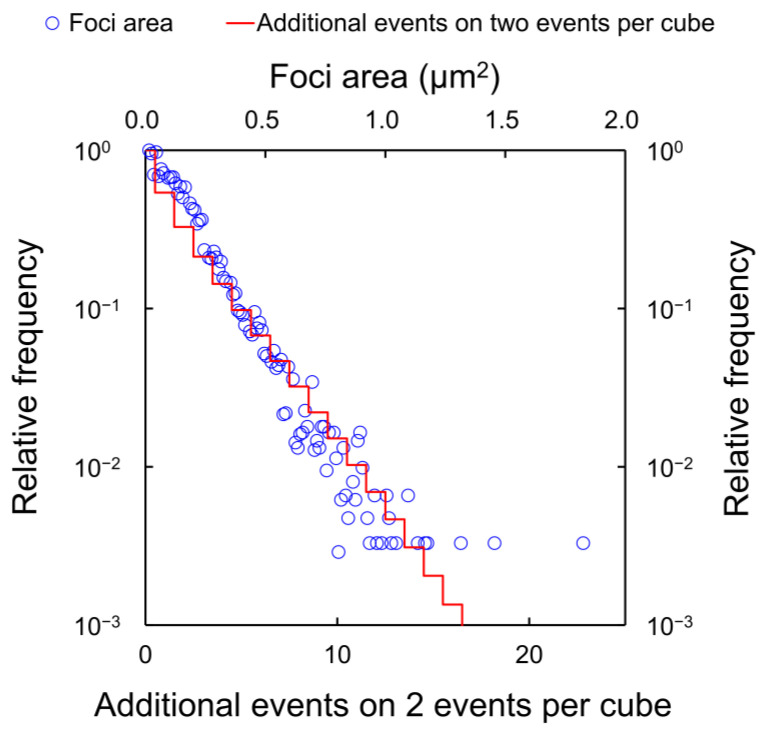
Relationship between the distribution of γ-H2AX foci area and additional events on two events per cube (called cluster-size). We compared the distribution of γ-H2AX foci area (experimental data) and the cluster size (calculation data by WLTrack) induced after irradiation with 35 kVp X-rays. As both distributions showed a similar tendency, the calculated distributions were fitted to the exponential data using least-square methods. The conversion coefficient of the horizontal axis from foci size to the number of events was determined to match both distributions and was found to be 13.2 ± 0.268 (/μm^2^) in this experimental system. There is a correlation between these distributions (*R*^2^ = 0.982).

Using the experimental foci data for 35 kVp X-rays and the corresponding estimation of cluster size, we obtained the relation between the relative frequency distributions of the foci area and the probability of cluster size for two events per cube, as shown in Figure 4. Note that we assumed that two events are required to induce a DSB from our previous model [18]. The distribution of events per cube at nm scale (i.e., 5.03 × 5.03 × 5.03 nm^3^) shows good agreement with the distribution of focus size. In the preliminary test, we calculated the number of events per cube for various cube sizes. Among the various sizes, the nm size of 5.03 × 5.03 × 5.03 nm^3^, which is the same volume as that in the sampling site used in the previous study by Garty et al. [34], showed the best agreement with the foci size distribution (see Figure 4). This agreement shows that the number of events in a cube is proportional to the foci size, and indicates that ionization and excitation can induce strand break. Assuming that the foci area is proportional to the cluster size, the conversion coefficient from the foci area to the cluster size was determined to be 13.2 μm^−2^ in the experimental distribution. Note that this conversion coefficient of horizontal axis from foci size to the number of events was determined to match both distributions by the least-square method, and there is a correlation between these distributions (*R*^2^ = 0.982). The standard deviation calculated by the error propagation was 0.268. From the good agreement of the distributions (Figure 4), DSB complexity can be estimated from the foci area detected by the γ-H2AX focus formation assay.

**Figure 5:**
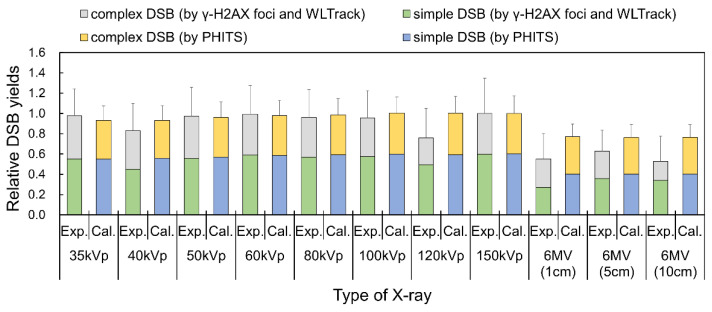
Fractions of DSB complexity determined by experiment and simulation. The fraction of simple and complex DSBs estimated by the number of γ-H2AX foci compared to the estimation by the DNA damage estimation model by the PHITS code. Note that the complex DSB is composed of DSB coupled with an SB (DSB+) and DSB coupled with 2 SBs (DSB++). Based on total yield of DSBs, it is suggested that the diagnostic X-rays exhibit higher biological impacts compared to the therapeutic X-rays.

Using the conversion coefficient determined above, we converted the threshold value for DSB complexity based on the cluster size to that based on the foci area. This threshold value was determined to reproduce the fraction of complex DSBs for 35 kVp X-ray calculated using the PHITS code. Based on this threshold, we estimated the fraction of isolated (simple) or complex DSBs using a γ-H2AX focus formation assay for various X-ray spectra. The DSB complexities evaluated by these methods were compared to the results estimated using the DNA damage estimation model in the PHITS code. The relative yields of simple and complex DSBs for various X-ray spectra are shown in Figure 5. As shown in Figure 5, the yields of simple and complex DSBs obtained from the foci area agreed well with those calculated by the PHITS code (*R*^2^ = 0.779). From this result, the developed method was found to be applicable to focus data measured for various X-ray spectral situations (i.e., diagnostic kV X-rays and therapeutic MV X-rays).

**Figure 6:**
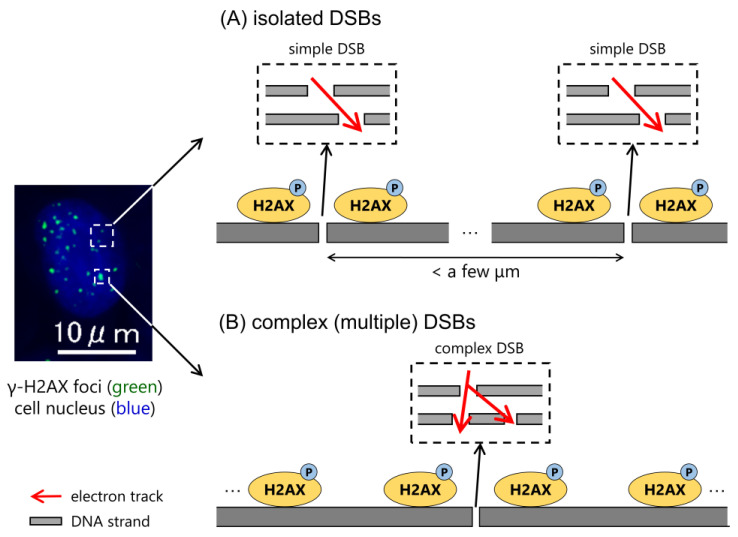
Schematic diagram of H2AX phosphorylation in isolated and complex (multiple) DSBs. In the case of low-LET radiation (i.e., X-ray), we assumed that the large foci reflect did not pile up some isolated DSBs (**A**) but a wide range of H2AX phosphorylation induced by the complex (multiple) DSBs (**B**). The degree of phosphorylation of H2AX was found to reflect the DSB complexity in nm scale.

From the simulation standpoint, the possibility to induce a DSB from a linkage (which is defined as the pair of two events within 10 bp (i.e., 3.4 nm)) is 0.00124 (DSB per linage). Considering this, DSBs can be induced at the track end of secondary electrons, and there are very few cases in which two or more DSBs occur simultaneously along an electron track. These facts suggest that the large foci reflect not piled up some foci composed of isolated DSBs (see Figure 6A), but a complex (or multiple) DSB (see Figure 6B). This suggestion is consistent with a previous report [11]. As the foci induced by low-LET radiation show various sizes, we assumed that the foci area was caused by one foci, including a large number of γ-H2AX. Therefore, as one of the mechanisms of γ-H2AX focus formation, during low-LET radiation (e.g., electrons and photons), it was found that the degree of phosphorylation of H2AX reflects the DSB complexity at the nm scale.

However, the yields of total DSBs for 35–150 kVp X-rays were higher than those for 6 MV-linac X-rays, indicating that diagnostic X-rays exhibit higher biological impacts than therapeutic X-rays. This tendency agrees well with what has been reported in previous studies [12,13]. Considering the biological effects of X-ray irradiation, it is necessary to note that the total number of DSBs (sum yield of isolated and complex DSBs) is a more important parameter than the DSB complexity. Note that the γ-H2AX foci area depends on the performance of the fluorescence microscope, such as resolution. When adopting this method in the analysis of cluster damage, it is necessary to set the optimum coefficient for the foci area obtained by the respective fluorescence microscope using the method. Currently, because the MC code for radiation transport, such as PHITS, is publicly available, it is possible for other researchers to calculate the conversion factor.

In this study, we focused on photon irradiation (as well as electron irradiation) and developed an analytical method for quantifying the DSB complexity using the γ-H2AX foci area. When discussing complex DNA lesions, detecting co-localized DSBs and non-DSBs (e.g., SSB and BD) [35], it is necessary to understand the biological impact of irradiation on cell-killing effects. In addition, as the γ-H2AX focus area induced after high-LET radiation (e.g., alpha particles and carbon ions) is larger than that of low-LET radiation (e.g., photons and electrons) [10,11,17], because high-LET radiation induced a large number of isolated foci along the track (see Figure 6A), and these foci are piled up. Further development of the estimation method is necessary so that it can be applied to such radiation. One of the limitations was that we calculated only physical processes such as the atomic interactions by electrons in liquid water. However, chemical processes for high-LET radiation (i.e., the diffusion and reaction of radical species) are also very important.

## 3. Materials and Methods

### 3.1. Cell Line and Cell Culture

We used a mammalian cell line, Chinese hamster lung fibroblast V79-379A, obtained from JCRB Cell Bank, Osaka, Japan (IFO50082). V79-379A cells were maintained in Minimum Essential Medium Eagle (M4655, Sigma, St. Louis, MO, USA) supplemented with 10% fetal bovine serum (Equitech-Bio Inc., Kerrville, TX, USA) and 1% penicillin/streptomycin (P4333, Sigma, St. Louis, MO, USA) at 37 °C in a humidified 95% air and a 5% CO_2_ incubator. The cells were seeded onto ϕ12-mm glass-based dishes (3911–035, IWAKI, Sayama-shi, Japan).

### 3.2. Irradiation Setup

We used various types of X-ray spectra: 35, 40, 50, 60, 60, 80, 100, 120, and 150 kVp with 1.6 mm Be and 1.0 mm Al filtration (MBR-1520R-4, Hitachi Power Solutions Co., Ibaraki, Japan) and 6 MV-linac (Varian 600 C linear accelerator, Varian Associates, Palo Alto, CA, USA). The mean X-ray energy for each X-ray spectra is also summarized in Table S1 in the Supplementary Material. The dose rates for 35, 40, 50, 60, 60, 80, 100, 120, and 150 kVp X-rays were 0.17, 0.22, 0.36, 0.49, 0.75, 1.02, 1.34, and 1.77 Gy/min, respectively. For the 35–150 kVp X-rays, the dose rate at targeting position was measured by the ion chamber (TN31013, PTW, Freiburg im Breisgau, Germany). The dose attenuation is negligible in the culture medium (the depth is 1 mm as water equivalent) for all types of X-rays. For the 6 MV linac X-rays, the dose rate was measured according to the Japanese Standard Dosimetry 12. The rates at the isocenter for in-field 6 MV X-rays at 1, 5, and 10 cm depths were 4.91, 4.44, and 3.75 Gy/min, respectively. For MV X-ray irradiation, the irradiated field size was 10 × 10 cm^2^, and the cell culture dishes were filled with the cell culture medium. An absorbed dose of 1.0 Gy was delivered to the cells for all types of X-rays. Each experiment was performed at room temperature.

### 3.3. Detection of DSBs by γ-H2AX Focus Formation Assay

Thirty minutes after irradiation, cells were fixed with 4% paraformaldehyde for 10 min. After rinsing with phosphate-buffered saline (PBS), the cells were permeabilized in ice-cold 0.2% Triton X-100 in PBS for 5 min and blocked with a solution of 1% bovine serum albumin (BSA) in PBS for 30 min. A primary antibody, γ-H2AX (ab26350, Abcam, Cambridge, UK) diluted at 1:400 with 1% BSA in PBS, was then fed and stored overnight at 4 °C. After rinsing thrice with 1% BSA in PBS, Alexa Fluor 594-conjugated goat anti-rabbit (ab150116, Abcam, UK), diluted at 1:250 with 1% BSA in PBS, was added and kept for 2 h. After rinsing thrice with 1% BSA in PBS, the cells were incubated with 1 μg/mL DAPI (62248, Thermo Fisher Scientific, Waltham, MA, USA) for 15 min. After rinsing once with methanol, the γ-H2AX foci were observed using a High Standard all-in-one fluorescent microscope (model BZ-9000; Keyence, Osaka, Japan).

First, we measured the distance between two γ-H2AX foci within a 5.0 μm diameter sphere in the cell nucleus. Second, we measured the area of the γ-H2AX foci. Third, we counted the number of γ-H2AX foci per cell nucleus to evaluate the dependency of X-ray energy on the relative biological effectiveness. All measurements were performed using ImageJ [36,37].

### 3.4. Calculation of Theoretical Data for the Distance between Two γ-H2AX Foci

The experimental distances between two γ-H2AX foci were compared to the theoretical distance between two points generated randomly in the circle as follows [38]:

(1) $$f\left(x,r\right)=\frac{4x}{{\pi r}^{2}}\cos^{-1}\left(\frac{x}{2r}\right)-\frac{{2x}^{2}}{{\pi r}^{3}}\sqrt{1-\frac{x^{2}}{{4r}^{2}}}$$

**Figure 7:**
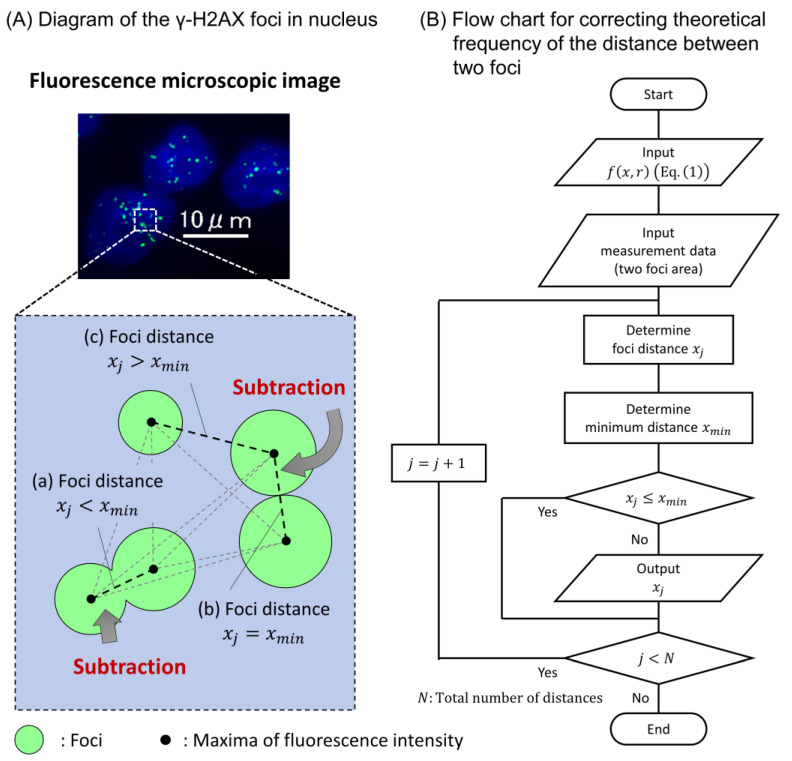
Representative illustration for measuring the distance between two points generated randomly in a site sphere. (**A**) shows the microscopic image of γ-H2AX foci, in which green dot and blue area represent DSB site and cell nucleus, respectively. We sampled the distance between two foci (as shown by (**c**)) except for that piled up with adjacent focus in such cases of (**a**,**b**). (**B**) shows the flow chart for correcting theoretical frequency of the distance between two foci.

The theoretical distribution was corrected by subtracting the frequency of the distances whose foci were piled up with the adjacent focus (Figure 7). First, we determined the arbitrary distance between two foci *x_j_* randomly using the input data *f*(*x*,*r*) (Equation (1)). Second, we calculated the minimum distance between two foci which were piled up by each other *x*_min_ by the foci area randomly extracted from the experimental data. In this calculation, the foci were assumed to be circular, and the distance data were subtracted when the distance of the foci was shorter than the minimum distance.

### 3.5. Simulation Setup

To estimate the yield of DSBs and the content of the complex form induced by photon irradiation, we used two Monte Carlo simulation codes: an in-house code for electron WLTrack [25] and the PHITS ver. 3.27 [26]. WLTrack and the *etsmode* [27,28] in PHITS are the event-by-event track-structure codes, which enabled us to calculate each atomic interaction (e.g., elastic scattering, ionization, and excitation) along electron tracks in liquid water. WLTrack has been well-validated for calculating the deposit energy within a microscale site (denoted as the scale of foci) and was adopted for estimating the number of inelastic events per focus. The *etsmode* has been well validated in previous studies [13,18] for sampling the inelastic interaction within nanoscale sites to estimate the yield of DSBs. The cut-off energy was set to be 1.0 eV for both codes.

### 3.6. Calculation of DNA Damage Complexity for X-ray Irradiation

**Figure 8:**
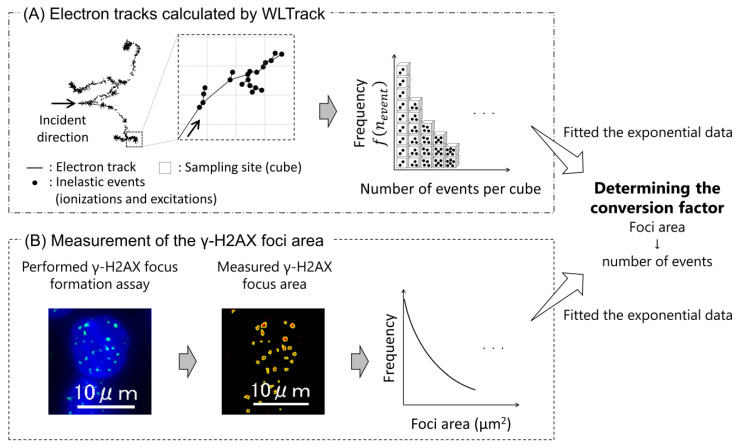
Sampling technique of the inelastic events along electron tracks and the estimation methods for the local density of inelastic events at DSB site. (**A**) shows 10 keV electron tracks calculated by the WLTrack code, in which the spatial coordinates of ionizations and excitations were calculated. Electron tracks pass into cubes, and the number of events per cube was sampled. (**B**) shows fluorescence microscopic image by the γ-H2AX focus formation assay and measurement of the foci area. In the left image of (**B**), green dot and blue area represent DSB site and cell nucleus, respectively. The green dot was detected by binarization processing, which is shown as red area in the central image of (**B**). After experiment (**A**) and calculation (**B**), both frequency of and the foci area and the number of events per cube (more than two events) was fitted to the exponential data, and the conversion coefficient from the foci area to the number of events was determined.

To evaluate the relationship between the foci area and inelastic event-cluster size, the local density of ionization and excitation was estimated based on the WLTrack code [25]. Assuming equilibrium of the secondary electrons, we sampled the initial spectrum for secondary electrons induced by X-ray irradiation in liquid water, which was calculated based on the electron gamma shower (EGS) mode [39] in the PHITS code. Note that the [t-product] tally was used for the sampling of secondary electrons, which is the estimator function that enables the calculation of the energy spectrum by counting secondary electrons induced by the atomic interaction between the X-rays and liquid water (i.e., photoelectric effect, Compton scattering, and pair production). The sampling cubes (i.e., 5.03 × 5.03 × 5.03 nm^3^), having a volume equivalent to that of the sampling cylinder used in a previous study [34], were randomly placed along electron tracks, and the number of inelastic events (i.e., ionizations and excitations) were scored per sampling cube (see Figure 8A). As we assumed that the number of events is proportional to the foci area, both the frequency of the number of events (see Figure 8A) and the foci area (see Figure 8B) were fitted to exponential data, and the conversion coefficient from the foci area to the number of events was determined. The frequency of foci area for 35 kVp X-rays was converted to the frequency of the number of events, and the threshold value of the number of events for classifying DSB complexity was determined to match the fraction of complex DSBs calculated by PHITS (whose estimation model is described in this section). Converting from the threshold of the number of events to the foci area, the fraction of complex DSB was estimated by the frequency of the foci area for various X-ray energies. From the number of DSBs induced X-ray irradiations counted by the γ-H2AX focus formation method, the yields of simple or complex DSBs were estimated using the fractions for various X-ray energies.

The DSB complexity estimated using the γ-H2AX focus formation assay and WLTrack was compared with that calculated using PHITS. The yield of the DSBs was estimated using the analytical code in PHITS version 3.27 [26]. In this model, the density of inelastic events calculated using the PHITS *etsmode* was measured [13,18]. To estimate the SB-induced local density of inelastic events, the number of event pairs (so-called linkage) within 3.4 nm (presumed to be the DSB site) per track *N*_link_ was scored. As previously reported [13,18], assuming that the number of linkages per track *N*_link_ per energy deposition of an electron track passing in liquid water *E*_dep_ is proportional to the DSB induction, we calculated the yield of DSBs (*Y*_DSB_) as follows:

(2) $$Y_{\mathrm{DSB}}{=k}_{\mathrm{DSB}}\frac{N_{\mathrm{link}}}{E_{\mathrm{dep}}}$$

where *k*_DSB_ is the proportionality constant (keV/Gy/Da). This DNA damage estimation model was in good agreement with experimental data and other simulations from a previous study [13]. Additionally, we estimated DSB complexity following a previous study [18]. In this model, we assumed that 12 inelastic events were needed on average to induce an additional strand break at a DSB site. The type of DSBs related to the DSB complexity was classified by the number of events (*N*_cl_) within a sampling site with a 10 bp radius (i.e., DSB site), namely, 2 ≤ *N*_cl_ < 14 for simple DSB, 14 ≤ *N*_cl_ < 26 for DSB+, and 26 ≤ *N*_cl_ < 38 for DSB++. Note that DSB+ is the DSB coupled with an SB within 10 bp and DSB++ is the DSB coupled with two SBs within 10 bp [3]. This model was in good agreement with the experimental data obtained using AFM [15].

For the X-ray source data, each X-ray spectrum calculated using the present formula was used as the input [40]. Then, the yield of the DSBs for the energies of secondary electrons induced by the X-ray spectra was calculated as follows [13]:

(3) $$\bar{Y_{*}}=\int Y_{*}\left(E_{\mathrm{dep}}\right)f\left(E_{\mathrm{dep}}\right){dE}_{\mathrm{dep}}$$

The photons were transported by the EGS mode [39] in PHITS, and secondary electrons induced by photon interactions were transported by *etsmode*. We estimated the fraction of simple or complex DSBs for irradiations with 35–150 kVp X-rays and 6 MV X-rays. The yield of DSBs was calculated with a large number of electrons to make the uncertainties less than a few percent in general.

## 4. Conclusions

In this study, we developed an analytical method for quantifying the DSB complexity induced by photon irradiation from microscopic images of γ-H2AX foci. Assuming that ionizations and excitations can induce DNA strand breaks (SBs), we scored the number of events in a water cube and the pair of events within a 10 bp separation along the electron track in order to estimate the yields of DSBs and the complexity. We then obtained the relationship between the events per cube (at a DSB site) and the focus area detected using a γ-H2AX focus formation assay. Using this relationship, we successfully reproduced the yields and fractions of complex DSBs induced by photon irradiation for various X-ray spectra. The developed method was found to be applicable to the foci data measured for various X-ray spectral situations.

To obtain the relationship between the foci area and the number of events at a DSB site, we used track-structure codes, i.e., in-house WLTrack codes for electrons. Results of the γ-H2AX focus formation assay (i.e., γ-H2AX foci distance and area) showed that the DSB complexity was independent of the X-ray energy. However, the number of DSBs per nucleus was different between diagnostic kVp X-rays and therapeutic 6 MV X-rays. These results indicate the total DSB yield (i.e., the isolated and complex DSB yields) to evaluate the biological impacts of photon irradiation. The present technique of evaluating DNA damage complexity would be beneficial for easily quantifying complex DSBs, but is limited only to photon and electron irradiation. In the case of low-LET irradiation, it is sufficient to analyze the spatial pattern of physical events [30]. When evaluating the DSB complexity for high-LET irradiation, chemical processes such as the yield of OH radicals are of importance [30]. In the future, it is necessary to further develop the technique for application to high-LET radiation.

## Data Availability

The data presented in this study are available in the article.

## Supplementary Materials

The following supporting information can be downloaded at: https://www.mdpi.com/article/10.3390/ijms24021386/s1.
