# Supplementary material for: An Analytical Method for Quantifying the Yields of DNA Double-Strand Breaks Coupled with Strand Breaks by γ-H2AX Focus Formation Assay Based on Track-Structure Simulation
Source: Int J Mol Sci. 2023 Jan 10;24(2):1386. doi: 10.3390/ijms24021386 (PMC9864015; doi:10.3390/ijms24021386)
Supplement: Supplementary file 1 [file ijms-24-01386-s001.zip › ijms-2114678-supplementary.pdf]

Supplementary data

An analytical method for quantifying the yields of DNA double-strand breaks coupled with strand breaks by γ-H2AX focus formation assay based on track-structure simulation

Yoshie Yachi, Yusuke Matsuya, Yuji Yoshii, Hisanori Fukunaga, Hiroyuki Date and Takeshi Kai

This supplementary data includes two figures and one table: Figure S1. Probability density of γ-H2AX foci area for various X-ray spectra used in this study; Figure S2. Energy spectra of photon and secondary electron for X-rays used in this study; Table S1. Average energy of photon and secondary electron generated by each X-ray spectra used in this study.

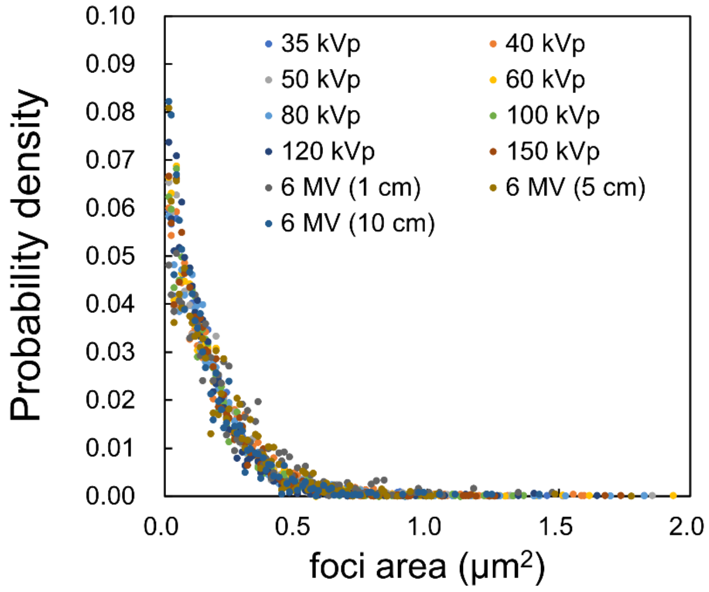

Figure S1. Probability density of γ-H2AX foci area for various X-ray spectra used in this study. In these energy spectra, the distributions show the same tendency. From these results, we presented the mean value for each irradiation condition as shown in Fig. 3 (see main paper).

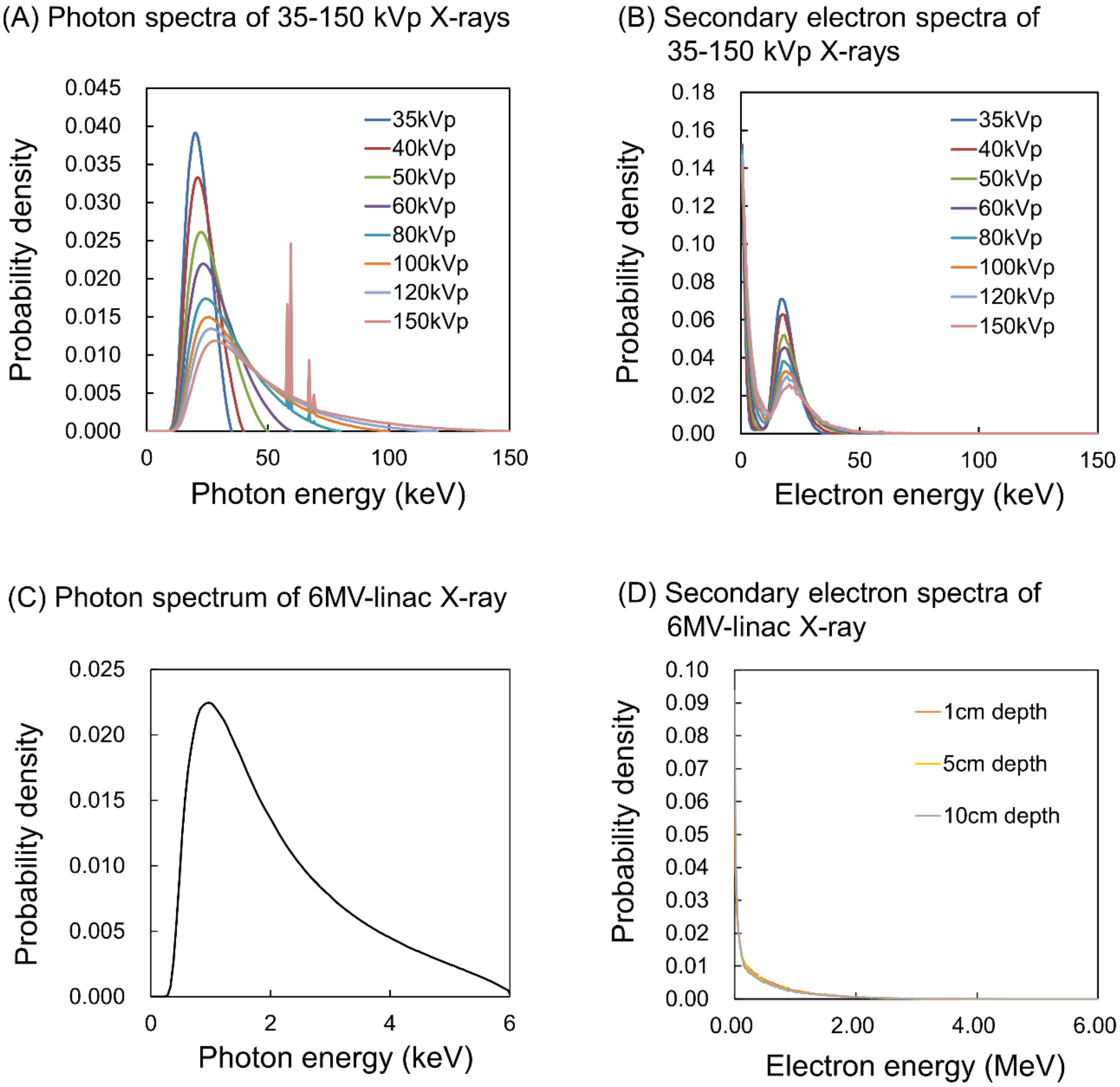

Figure S2. Energy spectra of photon and secondary electron for X-rays used in this study. These photon spectra were calculated using the present formula [1], and secondary electron spectra were calculated by the EGS mode [2] and *etsmode* [3,4] in PHITS [5].

Table S1. Average energy of photon and secondary electron generated by each X-ray spectra used in this study. These energies ware calculated by the probability density distributions as shown in Figure S2.

Type of X-ray	Average energy of photon (keV)	Average energy of electron (keV)
35 kVp X-ray (1 mm depth)	21.54	14.38
40 kVp X-ray (1 mm depth)	23.25	14.32
50 kVp X-ray (1 mm depth)	26.28	14.07
60 kVp X-ray (1 mm depth)	28.96	13.85
80 kVp X-ray (1 mm depth)	33.82	13.57
100 kVp X-ray (1 mm depth)	38.12	13.35
120 kVp X-ray (1 mm depth)	41.85	13.06
150 kVp X-ray (1 mm depth)	46.85	12.99
6 MV (1 cm depth at isocenter)	2070.09	530.04
6 MV (5 cm depth at isocenter)	2070.09	489.40
6 MV (10 cm depth at isocenter)	2070.09	476.69

References

1. Tucker, D. M.; Barnes, G. T.; Chakraborty, D. P. Semiempirical model for generating tungsten target x-ray spectra. Med. Phys. 1991, 18, 211-218.
2. Hirayama, H.; Namito, Y.; Bielajew, A. F.; Wilderman, S. J.; Nelson, W. R. The EGS5 code system. SLAC Report 730, prepared for the Department of Energy, USA (2005).
3. Kai, T.; Yokoya, A.; Ukai, M.; Watanabe, R. Cross sections, stopping powers, and energy loss rates for rotational and phonon excitation processes in liquid water by electron impact. Radiat. Phys. Chem. 2015, 108, 13–17.
4. Kai, T.; Yokoya, A.; Ukai, M.; Fujii, K.; Watanabe, R. Thermal equilibrium and prehydration processes of electrons injected into liquid water calculated by dynamic Monte Carlo method. Radiat. Phys. Chem. 2015, 115, 1–5.
5. Sato, T. et al. Features of Particle and Heavy Ion Transport code System (PHITS) version 3.02, J. Nucl. Sci. Technol. 2018, 55, 684-690.
